# ENSO Impact on Global Chikungunya Virus Transmission, 2008–2024: A Multi-Country Distributed-Lag Time-Series Analysis

**DOI:** 10.3390/v18080918

**Published:** 2026-08-21

**Authors:** Shi-Hui Shan, Long-Tao Chen, Wen-Qi Xie, Chen-Long Lv, Dong Jiang, Fang-Yu Ding, Gang Dong, Li-Qun Fang

**Affiliations:** 1 State Key Laboratory of Pathogen and Biosecurity, Academy of Military Medical Science, Beijing 100071, China; shanshihui666@163.com (S.-H.S.); chenlongtttt@163.com (L.-T.C.); tjjys2012@163.com (C.-L.L.); 2 Institute of Geographic Sciences and Natural Resources Research, Chinese Academy of Sciences, Beijing 100101, China; xiewenqi2523@igsnrr.ac.cn (W.-Q.X.); jiangd@igsnrr.ac.cn (D.J.); 3 College of Resources and Environment, University of Chinese Academy of Sciences, Beijing 100049, China

**Keywords:** chikungunya, El Niño–Southern Oscillation, distributed-lag time-series, climate change, CMIP6, disease projection

## Abstract

Chikungunya is undergoing global expansion, but how El Niño–Southern Oscillation (ENSO) influences its transmission remains unclear. We aim to assess the impact of ENSO phases on chikungunya incidence via temperature and precipitation teleconnections and to project future risk burden under climate change. We compiled annual national and subnational chikungunya case data (2008–2024), quantified ENSO–climate teleconnections using the E-index (eastern Pacific El Niño) and C-index (central Pacific La Niña), and applied distributed-lag time-series models to assess the teleconnection-mediated impact of ENSO on chikungunya incidence. We further projected future risk burden under climate change scenarios. Modelling shows that El Niño increased chikungunya risk after a 2-year lag (cumulative relative risk [CRR] = 1.40, 95% confidence interval [CI]: 1.02–1.85), while La Niña suppressed it (CRR = 0.09, 95% CI: 0.07–0.13). Temperature teleconnections were the dominant modifier of spatial heterogeneity in effects. Under all scenarios, El Niño-driven warming led to positive median excess cases, the highest under SSP2-4.5, whereas La Niña-driven changes projected smaller, highly uncertain reductions. ENSO influences chikungunya transmission through asymmetric and spatially heterogeneous teleconnection pathways. Integrating ENSO forecasts into surveillance efforts has the potential to enhance preparedness in climate-sensitive regions.

## 1. Introduction

Chikungunya, caused by the chikungunya virus (CHIKV) and transmitted primarily by *Aedes aegypti* and *Aedes albopictus*, typically causes fever and severe joint pain [1,2]. Since its identification in Tanzania in 1952, chikungunya has expanded across Africa, Asia, Europe, and the Americas [3]. By 2025, current or previous autochthonous transmission had been reported in 119 countries and territories, and several regions experienced reemerging outbreaks [4]. Annual global infections are estimated at 35 million [5], underscoring the need to identify climate-sensitive warning signals.

Chikungunya transmission is climate-sensitive, but reported climate associations vary by region [6]. Warm and dry conditions were linked to the 2004 East African coastal outbreak [7], whereas drought showed negative associations in Central Africa and Southeast Asia [8]. Extreme rainfall preceded local outbreaks in southern France [9], while temperature and precipitation were not significant in Colombia [10]. These contrasting findings indicate that broad climate drivers may act through region-specific pathways. ENSO is a major source of interannual climate variability. Its warm phase (El Niño) and cold phase (La Niña) alter temperature and precipitation across tropical and subtropical regions [11]. However, how ENSO teleconnections modulate chikungunya transmission, and whether temperature and precipitation pathways differ, remains insufficiently understood.

We therefore used the E-index and C-index to distinguish eastern Pacific El Niño and central Pacific La Niña events, quantified local temperature and precipitation teleconnections, and projected future ENSO-related chikungunya burden under climate-change scenarios.

## 2. Materials and Methods

### 2.1. Data Sources and Processing

We compiled annual chikungunya case data for 2008–2024 from public surveillance sources, including the Global Infectious Diseases and Epidemiology Online Network (GIDEON), the European Centre for Disease Prevention and Control (ECDC), and the published literature. To capture within-country regional heterogeneity, we additionally collected subnational surveillance data for the six largest countries by land area (China, the United States, Brazil, Australia, Canada, and India), obtained from their respective national disease surveillance systems. Russia was excluded because few chikungunya cases were reported. Detailed case data sources are provided in Supplementary Table S1. For model inputs, geographic units that only reported imported cases and units with more than three years of missing data were excluded; the remaining sporadic missing values were imputed using cubic spline interpolation. To improve comparability across distinct surveillance systems, all locations were processed under this unified framework, and incidence was standardized per 100,000 population.

Monthly mean Sea Surface Temperature (SST) from the Hadley Centre Global Sea Ice and Sea Surface Temperature (HadISST) Dataset was used to construct the E-index and C-index. ERA5 temperature and precipitation data were aggregated to administrative units as population-weighted means using GPW-v4 population data. Annual population denominators came from GlobPOP for 2008–2022 and WorldPop for 2023–2024. We assessed consistency between the two population datasets using their 2022 overlap, which showed a global aggregate discrepancy of only 0.18%. Incidence was expressed per 100,000 population. The Oxford Stringency Index (OSI) was included for 2020–2022 and set to zero outside that period.

For future risk projections, we used monthly SST, atmospheric temperature, and daily precipitation data spanning 1850–2099 from six Coupled Model Intercomparison Project Phase 6 (CMIP6) models (MIROC6, MIROC-ES2L, MPI-ESM1-2-HR, NorESM2-MM, CESM2-WACCM, CMCC-CM2-SR5) under four Shared Socioeconomic Pathway (SSP) scenarios: SSP1-2.6, SSP2-4.5, SSP3-7.0, and SSP5-8.5. All gridded data were re-gridded to a consistent spatial resolution through bilinear interpolation using Python (version 3.12.3) with the xarray package (version 2024.10.0). Future population projections were sourced from WorldPop, with annual projections interpolated using the nearest-neighbor method. All the data are provided in Supplementary Table S2.

### 2.2. ENSO Index Construction

Given the diversity of ENSO manifestations and the complexity of its dynamics, a single SST-based index is insufficient to distinguish between different event types. Following the method proposed by Takahashi [12], monthly E-index and C-index values were derived from linearly detrended SST anomalies over the period 1960–2024 according to the following equations:

(1) $$E=\left({\mathrm{PC}}_{1}-{\mathrm{PC}}_{2}\right)/\sqrt{2}$$

(2) $$C=\left({\mathrm{PC}}_{1}+{\mathrm{PC}}_{2}\right)/\sqrt{2}$$

where ${\mathrm{PC}}_{1}$ and ${\mathrm{PC}}_{2}$ are the two leading principal components obtained from empirical orthogonal function (EOF) decomposition of monthly SST anomalies within the domain 20° S–20° N, 140° E–80° W. Given that ENSO events typically peak in boreal winter, the annual E-index and C-index values are defined as the mean of monthly indices from December of the preceding year to February of the current year (DJF).

### 2.3. Teleconnection Derivation

We first standardised the monthly mean temperature and the total precipitation, then applied linear detrending to the monthly standardised anomalies of each variable. For each administrative unit *i*, partial correlation coefficients between the temperature and precipitation time series and the DJF E-index were computed within a 15-month window spanning from June of the preceding year to August of the current year, controlling for the other climate variable in each case. The resulting correlation series were subsequently smoothed using a 3-month rolling average. The smoothed coefficients for the month with the largest absolute value was then extracted and retained with their original signs, yielding the temperature-specific ($\tau^{E,T}$) and precipitation-specific ($\tau^{E,P}$) E-index teleconnection indices. The C-index teleconnections ($\tau^{C,T}$ and $\tau^{C,P}$) were derived using the same procedure applied to the DJF C-index.

### 2.4. Statistical Analysis

We utilized a distributed-lag time-series model to quantify the effects of Eastern Pacific El Niño (E-index) and Central Pacific La Niña (C-index) on chikungunya incidence and to examine how local temperature and precipitation teleconnections modulate these effects. The model was specified as follows:

(3) $$\begin{array}{ll} \ln\left(y_{i,t}+\delta \right)=\sum_{l=0}^{j} & [X_{t-l}\left(\beta_{1l}+\gamma_{1l}*\tau_{i}^{X,P}+\theta_{1l}*\tau_{i}^{X,T}\right)\\ & +X_{t-l}^{2}\left(\beta_{2l}+\theta_{2l}*\tau_{i}^{X,T}+\gamma_{2l}*\tau_{i}^{X,P}\right)]+\alpha \mathrm{OSI}+u_{i}+\epsilon_{i,t} \end{array}$$

where $y_{i,t}$ represents the chikungunya incidence in administrative unit *i* at year *t*, and $\epsilon_{it}$ is the normal random error. δ is a small value added to ensure that $y_{i,t}+\delta >0$ when $y_{i,t}=0$. ${\bar{X}}_{t-l}$ represents the annual E-index or C-index values at lag *l*, with $X_{t-l}^{2}$ being their quadratic terms to capture nonlinear effects. $\tau_{i}^{X,T}$ and $\tau_{i}^{X,P}$ are the temperature and precipitation teleconnection strengths in administrative unit *i*, respectively. $u_{i}$ represents the administrative-unit fixed effects, which were included to control for time-invariant differences in baseline incidence and persistent cross-regional variation in surveillance intensity. The OSI was included as a pandemic control variable.

The maximum lag was set to two years based on mosquito-borne disease biology and the previous literature [13,14]. Sensitivity analyses compared maximum lags from 0 to 5 years. Minimum cumulative case thresholds of 100, 500, and 1000 cases were assessed using adjusted R^2^ and the Bayesian Information Criterion.

To separately quantify the effects of El Niño and La Niña events, the cumulative relative risks (CRRs) were estimated at each ENSO intensity level relative to ENSO-neutral conditions (E-index = 0 or C-index = 0):

(4) $${\mathrm{CRR}}_{i\_X}\left(X=x\right)=\exp(\sum_{l=0}^{j}[x(\beta_{1l}+\theta_{1l}*{\tau^{X,T}}_{i}+\gamma_{1l}*{\tau^{X,P}}_{i})+x^{2}(\beta_{2l}+\theta_{2l}*{\tau^{X,T}}_{i}+\gamma_{2l}*{\tau^{X,P}}_{i}])$$

To isolate the contributions of temperature and precipitation teleconnection pathways, the pathway-specific CRRs were computed as:

(5) $${\mathrm{CRR}}_{i,X}^{T}\left(X=x\right)=\exp(\sum_{l=0}^{j}\left[x\theta_{1l}*{\tau^{X,T}}_{i}+x^{2}\theta_{2l}*{\tau^{X,T}}_{i}\right])$$

(6) $${\mathrm{CRR}}_{i,X}^{P}\left(X=x\right)=\exp(\sum_{l=0}^{j}\left[x\gamma_{1l}*{\tau^{X,P}}_{i}+x^{2}\gamma_{2l}*{\tau^{X,P}}_{i}\right])$$

where ${\mathrm{CRR}}_{i,X}^{T}$ and ${\mathrm{CRR}}_{i,X}^{P}$ respectively represent the cumulative relative risks via the temperature and precipitation teleconnection pathways. Moreover, 95% confidence intervals were derived from the 2.5th and 97.5th percentiles of the bootstrap distribution.

### 2.5. CMIP6 Model Selection and ENSO Projections

CMIP6 model simulations were screened according to the criteria established in previous studies [15]. Model performance was evaluated using the quadratic coefficient α of the ${\mathrm{PC}}_{1}$ and ${\mathrm{PC}}_{2}$ relationship. Models were retained when α < −0.17, corresponding to 50% of the observed value (α = −0.34). Monthly ENSO indices and teleconnection strengths were computed for both the historical (1960–2024) and projection (2026–2090) periods across all retained realisations. The two time periods have the same duration (65 years each). To avoid potential biases, teleconnections were calculated separately based on the simulated data from the historical period, denoted by $\tau_{i}^{X,T,\mathrm{hist}}$ and $\tau_{i}^{X,P,\mathrm{hist}}$, and from the projection period, denoted by $\tau_{i}^{X,T,\mathrm{ssp}}$ and $\tau_{i}^{X,P,\mathrm{ssp}}$. Let the teleconnections based on the observed data during the historical period be $\tau_{i}^{X,T,\mathrm{obs}}$ and $\tau_{i}^{X,P,\mathrm{obs}}$. The teleconnections used for the actual prediction during the projection period are given by

(7) $$\Delta \tau_{X,T}=\frac{\tau_{i}^{X,T,\mathrm{ssp}}-\tau_{i}^{X,T,\mathrm{hist}}}{\tau_{i}^{X,T,\mathrm{hist}}},\;\Delta \tau_{X,P}=\frac{\tau_{i}^{X,P,\mathrm{ssp}}-\tau_{i}^{X,P,\mathrm{hist}}}{\tau_{i}^{X,P,\mathrm{hist}}}$$

(8) $${\hat{\tau }}_{i}^{X,T,\mathrm{ssp}}=\tau_{i}^{X,T,\mathrm{obs}}+\tau_{i}^{X,T,\mathrm{hist}}\Delta \tau_{X,T},\;{\hat{\tau }}_{i}^{X,P,\mathrm{ssp}}=\tau_{i}^{X,P,\mathrm{obs}}+\tau_{i}^{X,P,\mathrm{hist}}\Delta \tau_{X,P}$$

### 2.6. Counterfactual Scenario Construction and Disease Burden Projections

To assess the impacts of global warming on the risk of chikungunya transmission through ENSO and teleconnection pathways, we constructed counterfactual scenarios assuming different future trends in the absence of global warming. Previous studies indicate that climate warming primarily alters amplitude variability in ENSO, as reflected in the standard deviations of time series [16,17]. For each simulation, we rescaled the original projected ENSO time series according to Equations (9) and (10) to obtain counterfactual future ENSO time series. The counterfactual future time series of the E-index and C-index ($X^{\mathrm{cf}}$) are given by

(9) $$\Delta \sigma_{X}=\frac{sd\left(X^{\mathrm{ssp}}\right)-sd\left(X^{\mathrm{hist}}\right)}{sd\left(X^{\mathrm{hist}}\right)}$$

(10) $$X^{\mathrm{cf}}=\frac{X^{\mathrm{ssp}}}{\left(1+\Delta \sigma_{X}\right)}$$

where $X^{\mathrm{hist}}$ and $X^{\mathrm{ssp}}$ represent historical and projected simulated ENSO series, sd (·) is the standard deviation (amplitude) of each ENSO time series, and $\Delta \sigma_{X}$ represents the change in ENSO amplitudes due to global warming. For counterfactual future teleconnections, we simply use the values based on the observed data during the historical period as calculated when fitting the models, i.e., $\tau_{i}^{X,T,\mathrm{cf}}=\tau_{i}^{X,T,\mathrm{obs}}$ and $\tau_{i}^{X,P,\mathrm{cf}}=\tau_{i}^{X,P,\mathrm{obs}}$, assuming teleconnections do not change in the absence of global warming.

After calculating the counterfactual future ENSO time series and teleconnections, we calculated the changes in chikungunya incidence in each administrative unit i and year t attributable to global warming according to the following equation:

(11) $$\begin{array}{c} \Delta y_{i,t}=\exp\left\{\sum_{L}^{j}\left[\begin{array}{c} \beta_{1L}{\bar{X}}_{t-L}^{\mathrm{ssp}}+\beta_{2L}{{\bar{X}}_{t-L}^{\mathrm{ssp}}}^{2}+\theta_{1L}{\bar{X}}_{t-L}^{\mathrm{ssp}}*{\hat{\tau }}_{i}^{X,T,\mathrm{ssp}}+\gamma_{1L}{\bar{X}}_{t-L}^{\mathrm{ssp}}*{\hat{\tau }}_{i}^{X,P,\mathrm{ssp}}+\\ \theta_{2L}{{\bar{X}}_{t-L}^{\mathrm{ssp}}}^{2}*{\hat{\tau }}_{i}^{X,T,\mathrm{ssp}}+\gamma_{2L}{{\bar{X}}_{t-L}^{\mathrm{ssp}}}^{2}*{\hat{\tau }}_{i}^{X,P,\mathrm{ssp}}+u_{i} \end{array}\right]\right\}-\\ \exp\left\{\sum_{L=0}^{j}\left[\begin{array}{c} \beta_{1L}{\bar{X}}_{t-L}^{\mathrm{cf}}+\beta_{2L}{{\bar{X}}_{t-L}^{\mathrm{cf}}}^{2}+\theta_{1L}{\bar{X}}_{t-L}^{\mathrm{cf}}*\tau_{i}^{X,T,\mathrm{cf}}+\gamma_{1L}{\bar{X}}_{t-L}^{\mathrm{cf}}*\tau_{i}^{X,P,\mathrm{cf}}+\\ \theta_{2L}{{\bar{X}}_{t-L}^{\mathrm{cf}}}^{2}*\tau_{i}^{X,T,\mathrm{cf}}+\gamma_{2L}{{\bar{X}}_{t-L}^{\mathrm{cf}}}^{2}*\tau_{i}^{X,P,\mathrm{cf}}+u_{i} \end{array}\right]\right\} \end{array}$$

Here, ${\bar{X}}_{t-L}^{\mathrm{ssp}}$ represents the average E-index and C-index values at the *l*th lag during the projection period under a given SSP scenario, and ${\hat{\tau }}_{i}^{X,T,\mathrm{ssp}}$ and ${\hat{\tau }}_{i}^{X,P,\mathrm{ssp}}$ denote the temperature and precipitation teleconnections, respectively. ${\bar{X}}_{t-L}^{\mathrm{cf}}$, $\tau_{i}^{X,T,\mathrm{cf}}$ and $\tau_{i}^{X,P,\mathrm{cf}}$ are the counterfactual counterparts in the absence of global warming, where teleconnections are assumed to remain at historical observed levels. To reduce the influence of extreme projections, log incidence values were trimmed using an interquartile-range treatment before exponentiation. This framework compares futures that differ in ENSO amplitude and teleconnection strength while holding other epidemiological factors constant.

## 3. Results

### 3.1. Global Distribution and Temporal Trends of Chikungunya Cases

**Figure 1:**
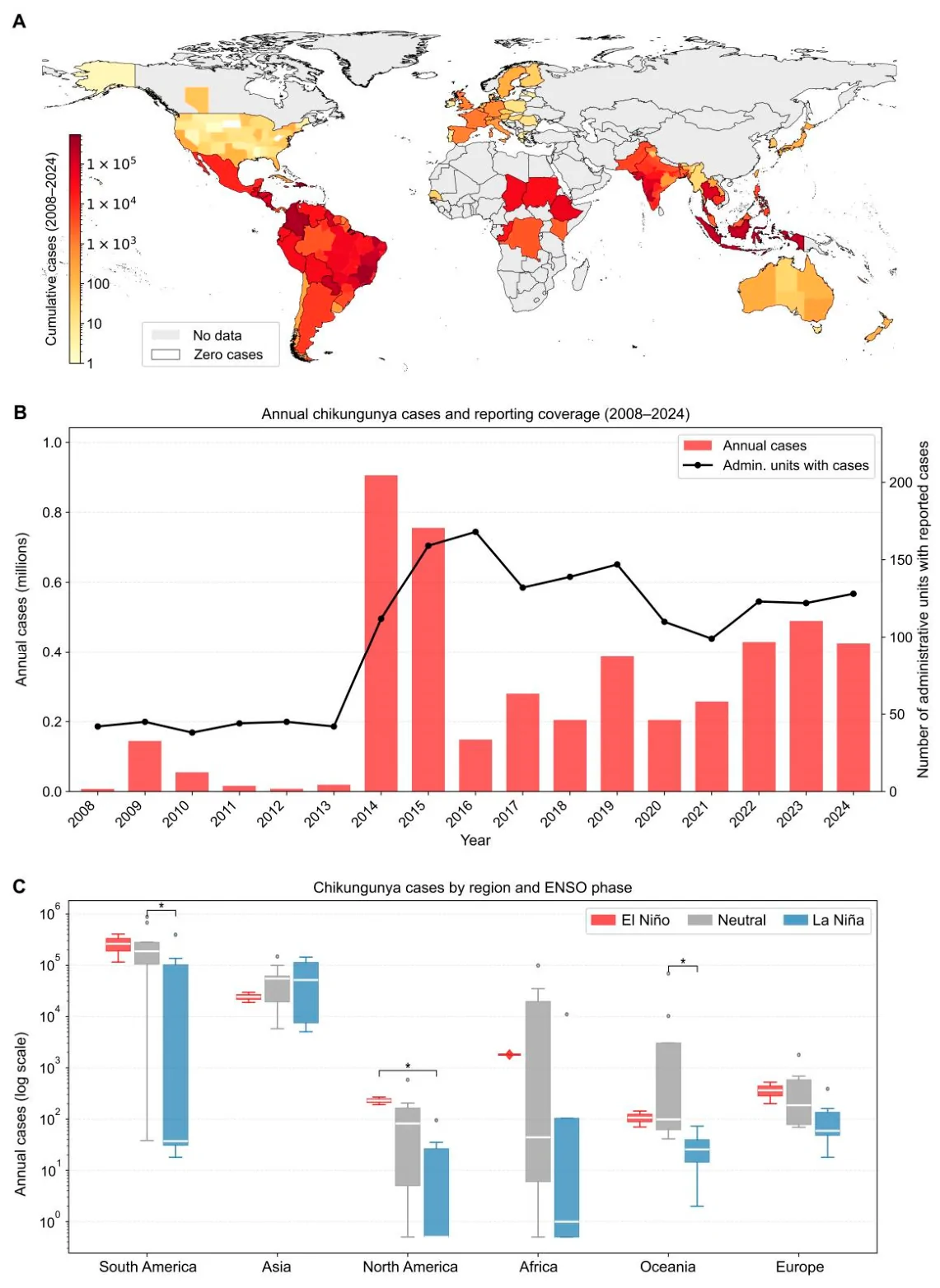
Global distribution and temporal trends of chikungunya cases from 2008 to 2024. (**A**) Spatial distribution of cumulative chikungunya cases at the national and subnational levels. Color gradient reflects the cumulative case counts on a logarithmic scale. Areas with grey background indicate regions where no surveillance data are available; areas with white background represent regions where surveillance has been confirmed but no cases were reported during the study period. (**B**) Annual totals of chikungunya cases (left axis, bars) and the number of nations or subnational areas with at least one reported case per year (right axis, line). (**C**) Annual case counts stratified by ENSO phase (El Niño, Neutral, La Niña) across six geographic regions. Each box represents the interquartile range; the horizontal line indicates the median; whiskers extend to 1.5 times the interquartile range; circles denote outliers. Diamond symbols indicate regions with only a single year of data for that ENSO phase. Y-axis is on a logarithmic scale. Asterisks (*) indicate statistically significant pairwise differences between ENSO phases.

During 2008–2024, a total of 5,007,738 cases of chikungunya were recorded across 230 administrative units, which included 108 nations and 122 subnational regions in our dataset, spanning all continents except Antarctica (Figure 1A). Cases were concentrated in South America, especially Brazil and neighboring countries, and in South and Southeast Asia. North America and Europe reported lower totals, mainly imported or travel-associated cases. Surveillance data were sparse in Africa, with only nine countries reporting cases.

Annual cases varied strongly over time. After the introduction of chikungunya into the Americas, the case counts reached a peak of approximately 0.94 million in 2014, followed by a secondary peak of 0.75 million in 2015 (Figure 1B). Subsequent to this outbreak period, the annual number of cases decreased but remained at a high level, ranging from 0.15 to 0.49 million between 2016 and 2024. Reporting units increased sharply during 2015–2016 and then stabilized.

Case counts differed by ENSO phase, but patterns varied across continents (Figure 1C). In South America, median case counts were significantly lower during La Niña years (37; IQR: 31–102,320) than during neutral years (186,895; IQR: 104,930–280,025) (*p* < 0.05). In North America, median case counts during El Niño years (230; IQR: 210–249) were significantly higher than during La Niña years (0; IQR: 0–26) (*p* < 0.05). In Oceania, median case counts were significantly lower during La Niña years (26; IQR: 15–40) than during neutral years (99; IQR: 62–3033) (*p* < 0.05). In Asia, La Niña years showed higher median case counts than El Niño years, whereas Africa and Europe showed higher median case counts in El Niño years; none of these differences reached statistical significance.

### 3.2. Impacts of ENSO on Local Temperature and Precipitation

**Figure 2:**
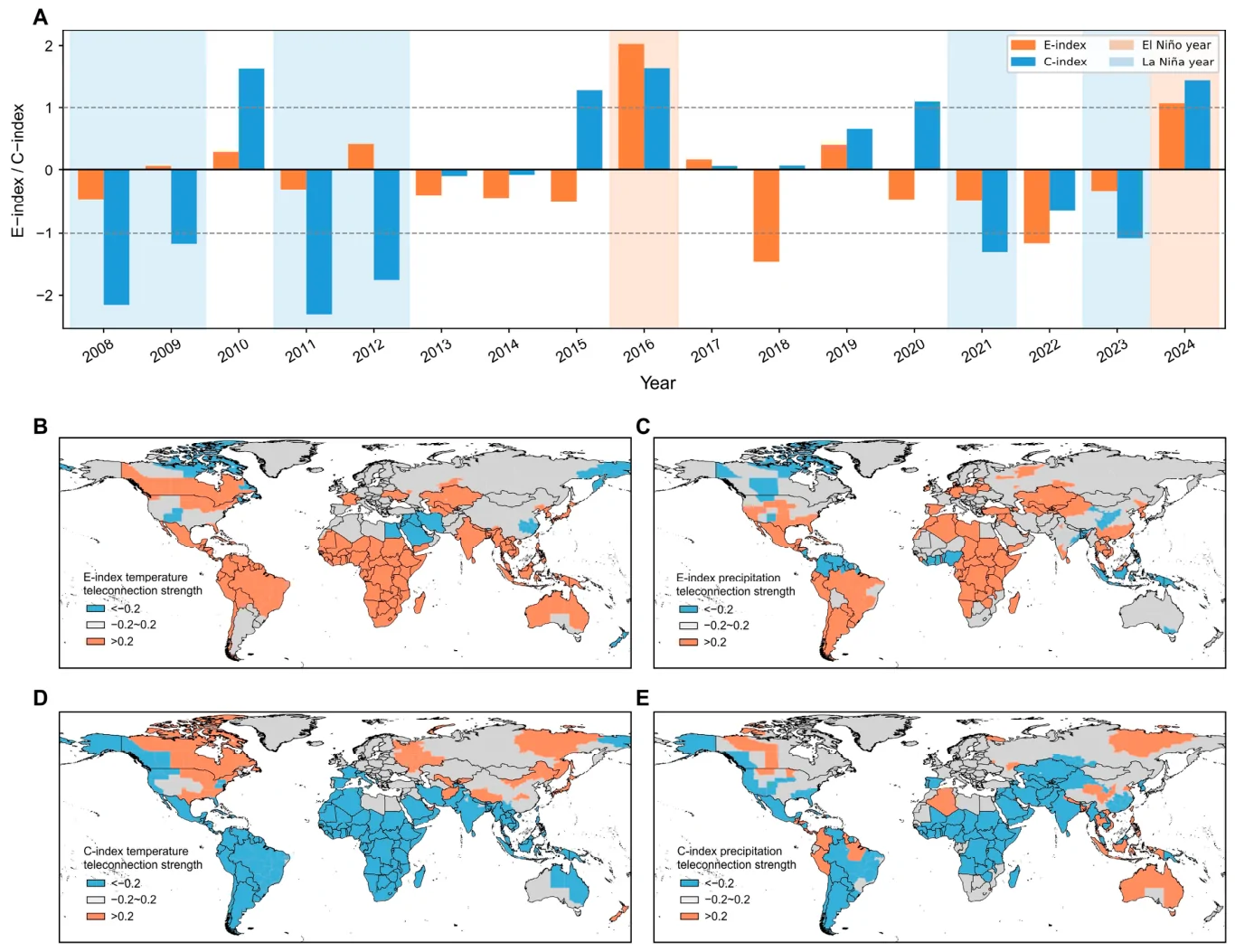
ENSO variability and global teleconnection patterns. (**A**) Annual standardised E-index (orange bars) and C-index (blue bars) during the study period from 2008 to 2024. Positive E-index values indicate El Niño conditions; negative C-index values indicate La Niña conditions. (**B**–**E**) Spatial distribution of partial correlation coefficients between the E-index and temperature (**B**), the E-index and precipitation (**C**), the C-index and temperature (**D**), and the C-index and precipitation (**E**) across all nations and subnational areas included in the analysis. Colours indicate the direction and strength of teleconnections: orange denotes positive correlations (>0.2), blue denotes negative correlations (<−0.2), and white denotes weak or non-significant correlations (−0.2–0.2).

The E-index and C-index displayed distinct interannual characteristics (Figure 2A). During the study period, one strong El Niño event (in 2016) and one moderate El Niño event (in 2024) were observed, along with three moderate La Niña events (2008–2009, 2011–2012, 2021–2023). Partial correlation analyses revealed that the E-index and C-index had spatially heterogeneous and asymmetric teleconnection impacts on local temperature and precipitation (Figure 2B–E). In general, the E-index and C-index exerted largely opposite effects on climate variables. For instance, the E-index was positively correlated with both precipitation and temperature across the majority of South America, the Caribbean Islands, Africa, and West and Central Asia, whereas in these regions, the C-index exhibited predominantly negative correlations with both precipitation and temperature. In addition, for both ENSO indices, temperature responses were more spatially extensive and relatively consistent, whereas precipitation responses exhibited greater regional heterogeneity and complexity.

### 3.3. Cumulative Relative Risk of ENSO for Chikungunya Incidence

Separate E-index and C-index models were fitted using a 1000-case inclusion threshold and a maximum 2-year lag. A total of 73 administrative units were included. Adjusted R^2^ was 0.54 for the E-index model and 0.46 for the C-index model (Supplementary Table S3). Lagged ENSO correlations were low, indicating no severe multicollinearity (Supplementary Tables S4 and S5).

**Figure 3:**
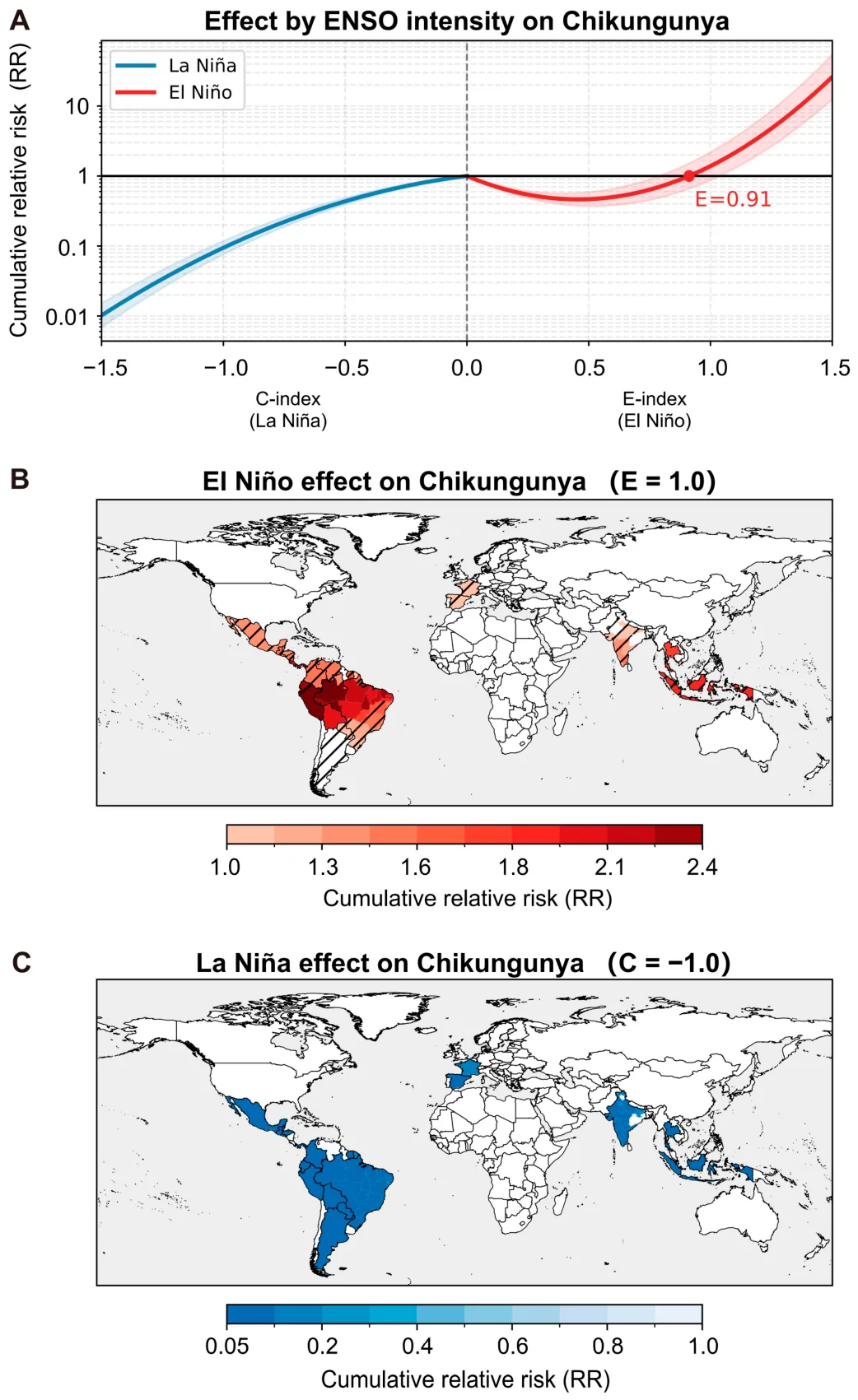
Global cumulative relative risk of ENSO on chikungunya incidence. (**A**) Dose–response curve showing the 2-year cumulative relative risk as a function of ENSO intensity. The red curve (right of the dashed line) represents El Niño (E-index > 0), and the blue curve (left) represents La Niña (C-index < 0). The filled circle marks the threshold E-index = 0.91. Shaded areas indicate 95% confidence intervals. (**B**) Spatial distribution of the 2-year cumulative relative risk of El Niño on chikungunya incidence at a fixed E-index value of 1.0. (**C**) Corresponding spatial distribution for La Niña at a fixed C-index value of −1.0. Color scales in panels B and C are truncated at the 97th percentile of unit-level cumulative RR values; units exceeding the upper bound are shown in the darkest shade.

El Niño showed a nonlinear association with chikungunya incidence (Figure 3A). When the E-index exceeded a threshold of 0.91, the cumulative relative risk was greater than 1.0; below this threshold, the association was negative. Among the 73 nations and subnational areas included in the analysis, 57 showed statistically significant positive cumulative effects (Figure 3B), with the most pronounced effect being observed in Peru, where the cumulative relative risk reached 3.65 (95% CI: 1.13 to 11.75). At E = 1.0, the cumulative effect increased with lag and reached 1.40 (95% CI: 1.02–1.85) at a 2-year lag (Supplementary Figure S1).

La Niña exhibited a significantly negative association with chikungunya incidence (Figure 3A). Under moderate La Niña conditions (C = −1.0), all 73 nations and subnational areas included in the analysis showed statistically significant suppression (Figure 3C). The strongest suppressive effect was observed in Nicaragua, where the cumulative relative risk was 0.04 (95% CI: 0.02–0.08). This effect also exhibited cumulative lag characteristics, reaching a maximum suppressive value of 0.09 (95% CI: 0.07–0.13) at a 2-year lag (Supplementary Figure S2).

Sensitivity analyses supported a 2-year maximum lag and the 1000-case inclusion threshold, which improved model fit and reduced instability from low-incidence regions (Supplementary Figures S1 and S2; Supplementary Table S3).

### 3.4. ENSO Influences the Risk of Chikungunya by Modulating the Local Temperature and Precipitation Conditions

**Figure 4:**
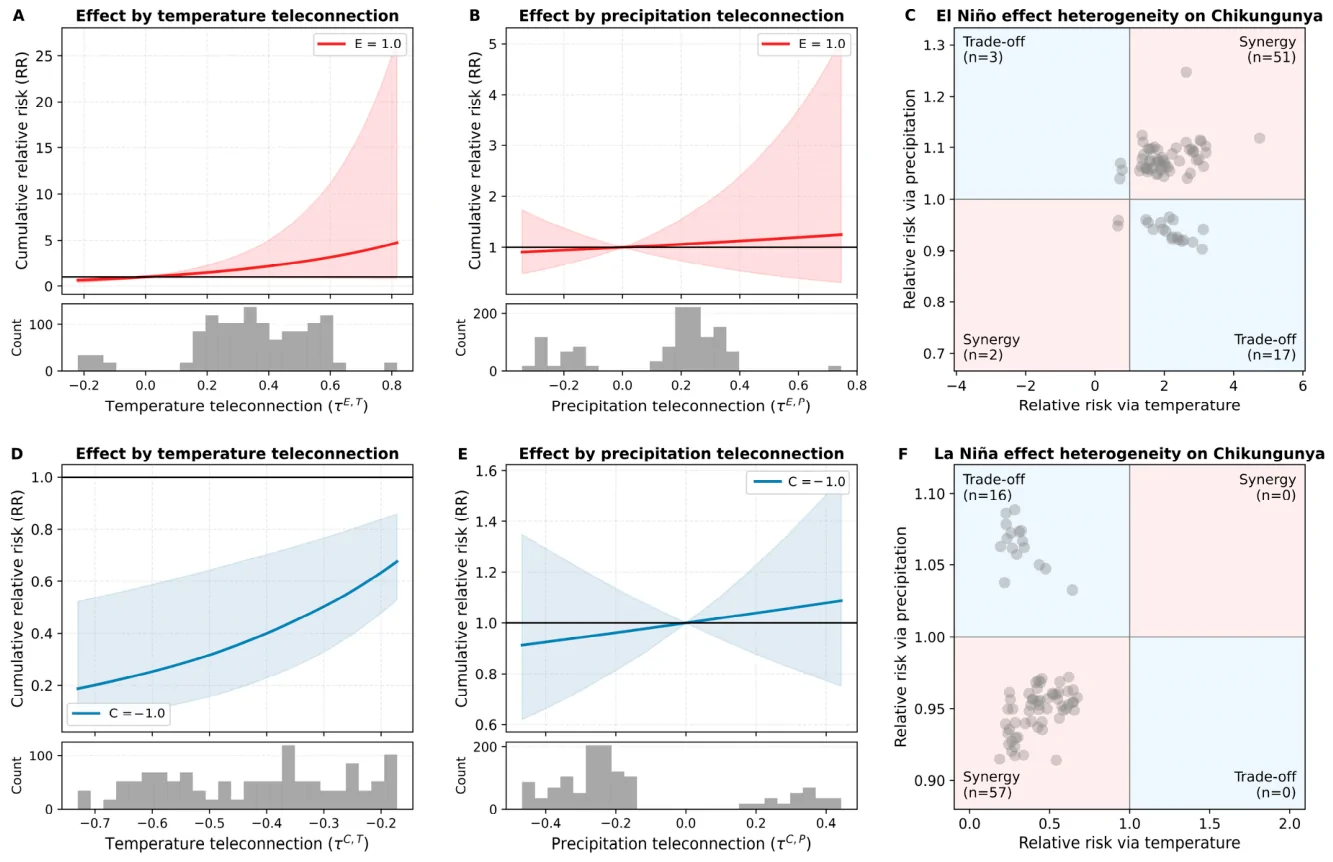
Persistent impact of ENSO on Chikungunya incidence through teleconnections. (**A**,**B**) 2-year cumulative relative risk via the temperature pathway (**A**) and precipitation pathway (**B**) as a function of teleconnection strength, evaluated at moderate El Niño (E = 1.0) intensities. Histograms show the distribution of teleconnection strength across nations and subnational areas. (**C**) Scatter plot of pathway-specific cumulative relative risks via temperature (*x*-axis) and precipitation (*y*-axis) for each nation and subnational area under moderate El Niño conditions (E = 1.0); the four quadrants correspond to synergistic and antagonistic patterns. (**D**,**E**) 2-year cumulative relative risk via the temperature pathway (**D**) and precipitation pathway (**E**) as a function of teleconnection strength, evaluated at moderate La Niña (C = −1.0). (**F**) Scatter plot of pathway-specific cumulative relative risks via temperature (*x*-axis) and precipitation (*y*-axis) for each administrative unit under moderate La Niña conditions (C = −1.0). Shaded areas in all panels indicate 95% confidence intervals.

The spatial heterogeneity in El Niño effects was more closely related to temperature than precipitation teleconnections. At E-index = 1.0, CRR increased with temperature teleconnection strength, although the trend was not statistically significant, whereas the precipitation pathway showed a weaker, non-significant association (Figure 4A,B). Among 73 administrative units, 51 showed increased risk through both pathways, 20 showed opposing pathway effects, and two showed suppression effects through both pathways (Figure 4C).

For La Niña, temperature teleconnections significantly modified risk: at C-index = −1.0, stronger negative temperature teleconnections were associated with greater suppression (Figure 4D). Precipitation teleconnections were not significant (Figure 4E). 57 administrative units showed decreased risk through both pathways, 16 showed suppressive temperature but promotive precipitation effects, and none showed positive contributions through both pathways (Figure 4F).

Excluding 2020–2022 produced coefficient patterns consistent with the primary model, supporting robustness to COVID-19-related surveillance disruption (Supplementary Tables S6 and S7).

### 3.5. The Projection of Future Risk Burden of Chikungunya Under Climate Change

After amplitude screening, 180 CMIP6 realizations were retained across the four SSP scenarios for future prediction (Supplementary Table S8). ENSO amplitude and teleconnection strength generally increased under future scenarios but with substantial inter-model variation (Supplementary Figures S3 and S4). Specifically, the median increase in El Niño amplitude ranged from 2.14–12.53% across scenarios, with temperature teleconnection increases of 3.11–10.10% and precipitation teleconnection increases of 5.28–10.44%. For La Niña, the median amplitude increase ranged from 6.92–9.25%, with temperature and precipitation teleconnection increases of 3.60–8.79% and 3.81–7.57%, respectively. Overall, the low-emission scenario (SSP1-2.6) was associated with the smallest projected changes in ENSO characteristics, while intermediate and high-emission scenarios generally showed larger increases in amplitude or teleconnection strength, although the pattern was not strictly monotonic.

**Figure 5:**
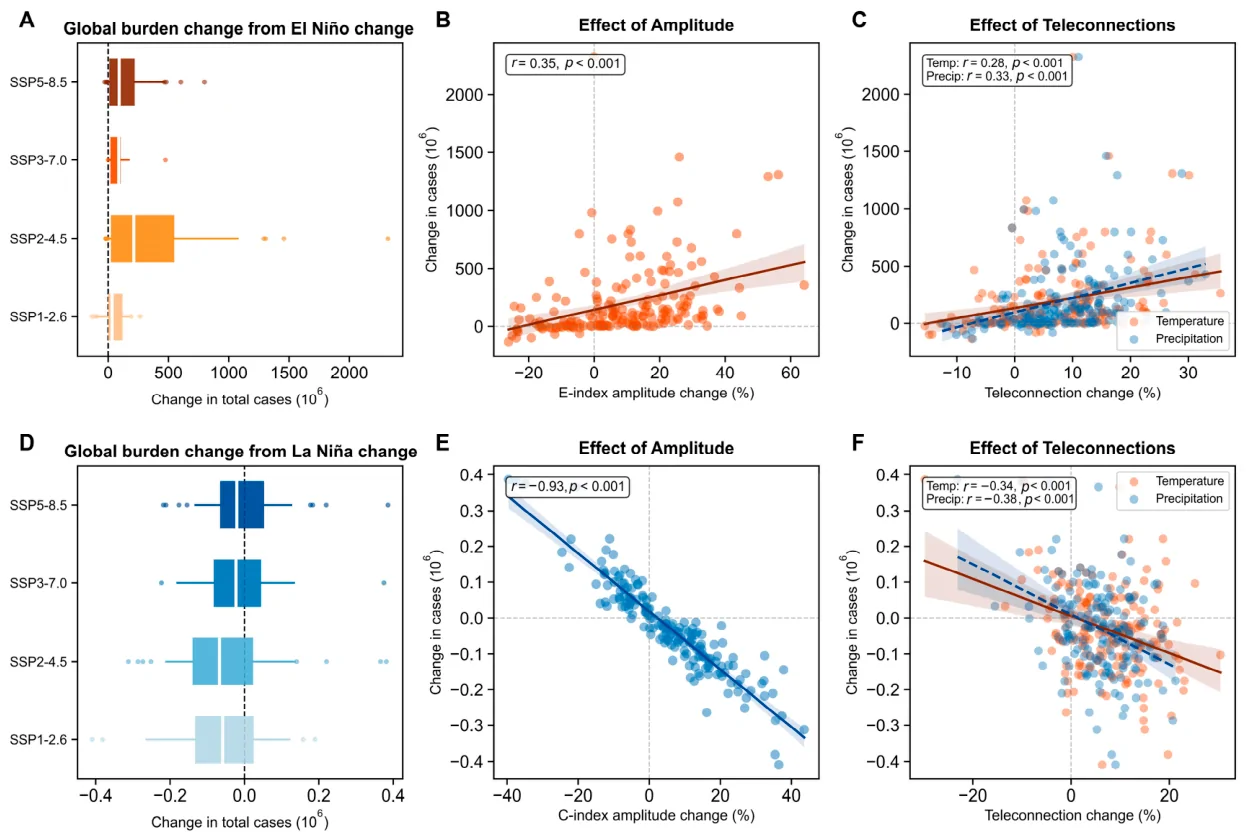
Projected chikungunya burden under future ENSO changes. (**A**,**D**) Box plots of projected changes in cumulative global chikungunya cases attributable to El Niño (**A**) and La Niña (**D**) characteristic changes driven by global warming under four SSP scenarios (SSP1-2.6, SSP2-4.5, SSP3-7.0, and SSP5-8.5). The white vertical line denotes the median, boxes span the interquartile range (Q1–Q3), whiskers indicate the 5th and 95th percentiles, and points beyond the whiskers represent outliers. (**B**,**E**) Associations between the projected percentage change in El Niño amplitude (E-index; (**B**)) and La Niña amplitude (C-index; (**E**)) and the corresponding projected change in total chikungunya cases (×10^6^). (**C**,**F**) Associations between projected percentage changes in temperature (red dots) and precipitation (blue dots) teleconnection strengths and the projected change in total chikungunya cases (×10^6^) for El Niño (**C**) and La Niña (**F**). Shaded bands indicate 95% confidence intervals. Each point represents one CMIP6 climate model simulation pooled across all four SSP scenarios.

Warming-induced El Niño changes produced positive median excess cases under all scenarios (Figure 5A). The largest median increase occurred under SSP2-4.5 (212.87 million), with wide realization-level uncertainty. This was followed by SSP5-8.5 and SSP3-7.0, whereas SSP1-2.6 showed the smallest increase (median 32.31 × 10^6^, ranging from −106.41 × 10^6^ to 208.18 × 10^6^ across realizations). Increases in projected E-index amplitude were positively associated with larger increases in case numbers (*r* = 0.35, *p* < 0.001), as were increases in temperature (*r* = 0.28, *p* < 0.001) and precipitation (*r* = 0.33, *p* < 0.001) teleconnection strength (Figure 5B,C).

Warming-induced La Niña changes were projected to reduce chikungunya cases but by far less than El Niño-related increases (Figure 5D). Median reductions ranged from 20,000 to 70,000 cases, with the most substantial reduction projected under SSP2-4.5. There was a strong negative association between the increases in C-index amplitude and case changes (*r* = −0.93, *p* < 0.001). Similarly, increases in the teleconnection strength of temperature (*r* = −0.34, *p* < 0.001) and precipitation (*r* = −0.38, *p* < 0.001) were also negatively correlated with case changes (Figure 5E,F). Projected changes were concentrated in South Asia, especially Maharashtra, Karnataka, and Tamil Nadu, with larger El Niño-related increases than La Niña-related reductions (Supplementary Figures S5 and S6).

## 4. Discussion

In this study, we used a distributed-lag time-series model to explore the cumulative and lagged associations between ENSO indices and chikungunya incidence at the national and subnational levels from 2008 to 2024. We quantified the spatial heterogeneity of ENSO effects through temperature and precipitation teleconnection metrics and projected the potential impact of future ENSO changes on global chikungunya burden under a warming climate using the CMIP6 multi-model ensemble. Previous studies have reported associations between ENSO and mosquito-borne diseases such as dengue and malaria [18,19]. However, quantitative studies specifically concentrating on chikungunya are still limited [13].

We found a significant positive association between El Niño and chikungunya incidence, with the 2-year cumulative relative risk reaching 1.40 (95% CI: 1.02 to 1.85). In contrast to previous studies using the Niño3.4 index that reported non-significant associations [20], our E-index and C-index models identified a significant positive association between El Niño and chikungunya incidence. This finding is consistent with previous reports regarding the lagged effects of ENSO on mosquito-borne diseases [21,22]. Notably, we identified a nonlinear threshold of E-index = 0.91. Below this threshold, El Niño was negatively associated with incidence, and above it, the association turned positive. This may reflect the nonlinear thermal response of *Aedes* mosquitoes and chikungunya transmission to ENSO-related climate anomalies [23,24]. La Niña showed the opposite pattern, with a significant negative association and a 2-year cumulative relative risk of 0.09 (95% CI: 0.07–0.13). La Niña events are typically associated with below-normal temperatures and precipitation anomalies in certain tropical regions, which may be unfavorable for the growth and reproduction of the *Aedes* population, and thus are likely to reduce incidence of chikungunya [8,25,26].

Regarding teleconnection pathways, it was found that temperature teleconnections exerted a more pronounced modulating effect on ENSO-associated chikungunya risk compared to precipitation teleconnections. This agrees with evidence that temperature directly affects mosquito development, survival, biting frequency, and viral replication, whereas precipitation acts more indirectly through breeding habitats and local hydrology [27]. The synergy-antagonism analysis also showed that temperature and precipitation effects can reinforce or offset one another across regions.

Future projections suggested that increases in El Niño amplitude and teleconnection strength under all four SSP scenarios were associated with higher chikungunya burden. This is consistent with dengue studies projecting that climate warming may amplify ENSO-related disease risk [27]. Notably, the median number of projected additional cases was highest under SSP2-4.5 (212.87 million cases) rather than under the highest emission scenario SSP5-8.5, which is consistent with projections in some studies that ENSO variability increases most substantially under intermediate-emission scenarios [28]. By contrast, changes in La Niña characteristics were associated with fewer cases, although confidence intervals crossed zero across all scenarios, highlighting the substantial uncertainty in future ENSO projections. With El Niño expected to strengthen through 2026, enhanced CHIKV surveillance and targeted vector control should be prioritized in the high-risk regions identified by our projections, particularly during the subsequent two years.

Several limitations should be acknowledged when interpreting these results. First, case data came from multiple public surveillance sources with heterogeneous reporting definitions, diagnostic capacity, and completeness. Although we harmonized records, excluded imported-only and highly incomplete units, conducted sensitivity analyses, and applied administrative-unit fixed effects to reduce bias resulting from persistent cross-location differences in baseline reporting, between-country and temporal surveillance heterogeneity including differences in diagnostic accessibility and residual underreporting, particularly in regions with sparse surveillance infrastructure, may still influence the risk estimates. Future studies could explicitly model surveillance uncertainty using disease-specific reporting probabilities or latent-variable approaches (e.g., Bayesian hierarchical models) to adjust for heterogeneous reporting rates. Second, the projections presented in this study were designed to isolate ENSO-related climatic changes and therefore did not dynamically incorporate multiple non-climatic determinants of chikungunya transmission and hold them constant, e.g., vaccination, urbanization, vector-control interventions, and temporal changes in population susceptibility. Natural CHIKV infection can induce long-lasting protective immunity [29], which indicates that large-scale outbreaks may reduce the size of the susceptible population independently of climatic conditions, while births and population migration may subsequently replenish susceptibility over time. Future studies that integrate SSP-consistent socioeconomic trajectories, seroprevalence data and vaccination scenarios will enable more comprehensive estimate of future incidence burden of chikungunya. Third, our macro-scale statistical framework does not explicitly represent biological heterogeneity at the vector and pathogen levels. Differences in ecological characteristics and vector competence between *Aedes aegypti* and *Aedes albopictus*, as well as the co-circulation of other *Aedes*-borne viruses and potential within-vector interactions, may partly account for the residual spatial and temporal variability [30]. Future work could link this framework with mechanistic biological models that include temperature-dependent mosquito traits and viral extrinsic incubation periods [22,24], together with regional climate model downscaling, to improve localized forecasting performance. In addition, structural uncertainty remains in future ENSO projections because CMIP6 models exhibit systematic differences in their representation of ENSO dynamics [28,31].

## 5. Conclusions

This study demonstrates that ENSO influences chikungunya virus transmission through nonlinear, asymmetric, and spatially heterogeneous teleconnection pathways, with effects accumulating over a period of up to two years. El Niño above the identified intensity threshold was associated with increased chikungunya risk, whereas La Niña generally showed a suppressive association, and temperature teleconnections played a more prominent role than precipitation teleconnections in modifying regional effects. Under future climate-change scenarios, the strengthening of El Niño amplitude and teleconnections may substantially increase the chikungunya burden, while the projected reductions associated with La Niña are smaller and more uncertain. Integrating ENSO and regional teleconnection forecasts into risk-based surveillance, early-warning systems, and targeted vector-control strategies may therefore improve preparedness for chikungunya outbreaks in climate-sensitive regions.

## Data Availability

Publicly available datasets were analyzed in this study; the corresponding data sources and URLs are provided in Supplementary Tables S1 and S2. The processed data supporting the findings of this study are available from the corresponding author upon reasonable request.

## Supplementary Materials

The following supporting information can be downloaded at: https://www.mdpi.com/article/10.3390/v18080918/s1, Figure S1: Sensitivity analysis of the cumulative lagged effects of El Niño (when E-index = 1.0) on chikungunya incidence under alternative maximum lag specifications; Figure S2: Sensitivity analysis of the cumulative lagged effects of La Niña (when C-index = −1.0) on chikungunya incidence under alternative maximum lag specifications; Figure S3: Projected changes in ENSO amplitude and teleconnection strength across SSP scenarios (2026–2090 relative to 1960–2024); Figure S4: E-index and C-index sum across scenarios; Figure S5: Projected additional chikungunya burden attributable to climate change-driven shifts in ENSO amplitude under four SSP scenarios (2030–2050); Figure S6: Projected averted chikungunya burden attributable to climate change-driven shifts in ENSO amplitude under four SSP scenarios (2030–2050); Table S1: Chikungunya case data sources; Table S2: Climate and Socioeconomic Data sources; Table S3: Comparison of model performance at different case thresholds under a maximum lag of two years; Table S4: Correlation matrix for the E-index and its lags; Table S5: Correlation matrix for the C-index and its lags; Table S6: Comparison of E-index model results accounting for the impact of the COVID-19 pandemic; Table S7: Comparison of C-index model results accounting for the impact of the COVID-19 pandemic; Table S8: CMIP6 models and realizations selected for predicting future infectious disease risks for each of the four Shared Socioeconomic Pathways scenarios (SSP1-2.6, SSP2-4.5, SSP3-7.0 and SSP5-8.5).

## Abbreviations

BIC	Bayesian Information Criterion
C-index	Central Pacific La Niña index
CHIKV	Chikungunya virus
CI	Confidence interval
CMIP6	Coupled Model Intercomparison Project Phase 6
COVID-19	Coronavirus disease 2019
CRR	Cumulative relative risk
DJF	December–January–February
ECDC	European Centre for Disease Prevention and Control
E-index	Eastern Pacific El Niño index
ENSO	El Niño–Southern Oscillation
EOF	Empirical orthogonal function
ERA5	Fifth generation ECMWF Reanalysis
GIDEON	Global Infectious Diseases and Epidemiology Online Network
GlobPOP	Global gridded population dataset
GPW-v4	Gridded Population of the World, Version 4
HadISST	Hadley Centre Global Sea Ice and Sea Surface Temperature dataset
IQR	Interquartile range
OSI	Oxford Stringency Index
SSP	Shared Socioeconomic Pathway
SST	Sea surface temperature
UI	Uncertainty interval
WorldPop	World Population project dataset
