# Supplementary material for: ENSO Impact on Global Chikungunya Virus Transmission, 2008–2024: A Multi-Country Distributed-Lag Time-Series Analysis
Source: Viruses. 2026 Aug 21;18(8):918. doi: 10.3390/v18080918 (PMC13517555; doi:10.3390/v18080918)
Supplement: Supplementary file 1 [file viruses-18-00918-s001.zip › viruses-4497251-supplementary.pdf]

Supplementary Material

ENSO impact on global chikungunya virus transmission, 2008–2024: a multi-country distributed-lag time-series analysis

Supplementary Figures and Tables

This file contains the following figures and tables:

Figures S1–S6

Tables S1–S8

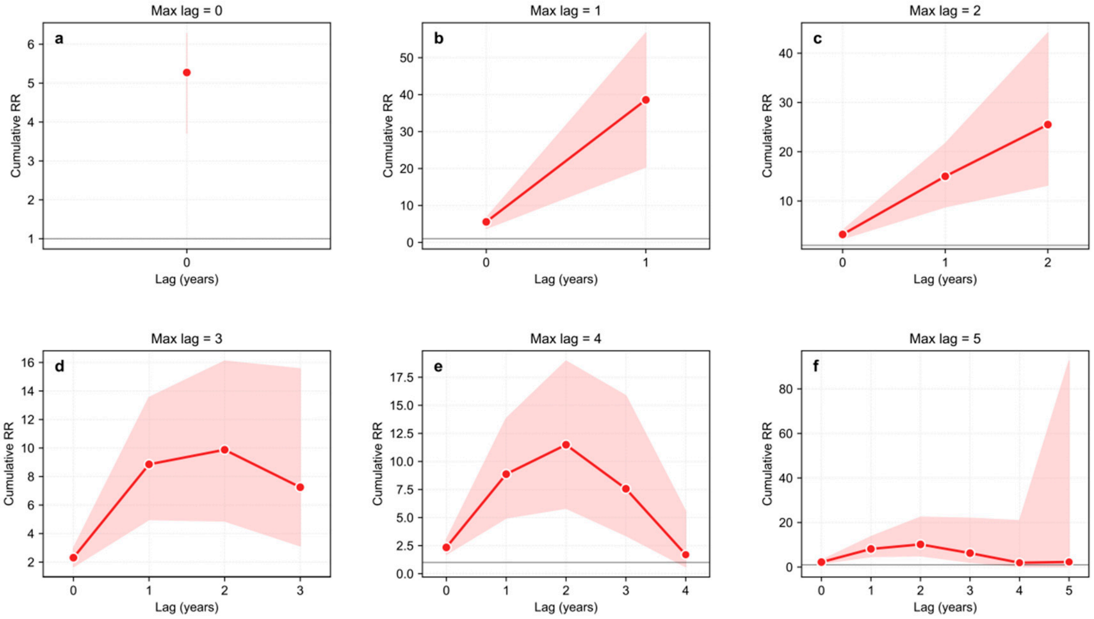

Figure S1. Sensitivity analysis of the cumulative lagged effects of El Niño (when E-index = 1.0) on chikungunya incidence under alternative maximum lag specifications. Each panel (a–f) shows the cumulative marginal effect estimated from models with a maximum lag of 0 through 5 years, respectively, evaluated at a fixed E-index value of 1.0. Error bars indicate 95% confidence intervals.

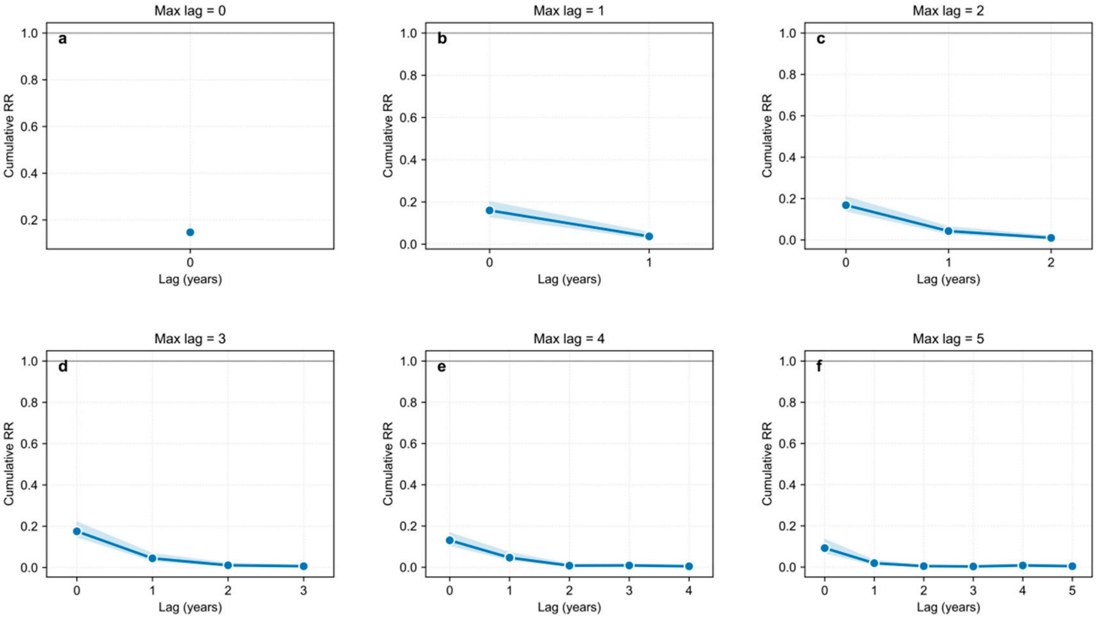

Figure S2. Sensitivity analysis of the cumulative lagged effects of La Niña (when C-index = −1.0) on chikungunya incidence under alternative maximum lag specifications. Each panel **(a–f)** shows the cumulative marginal effect estimated from models with a maximum lag of 0 through 5 years, respectively, evaluated at a fixed C-index value of −1.0. Error bars indicate 95% confidence intervals.

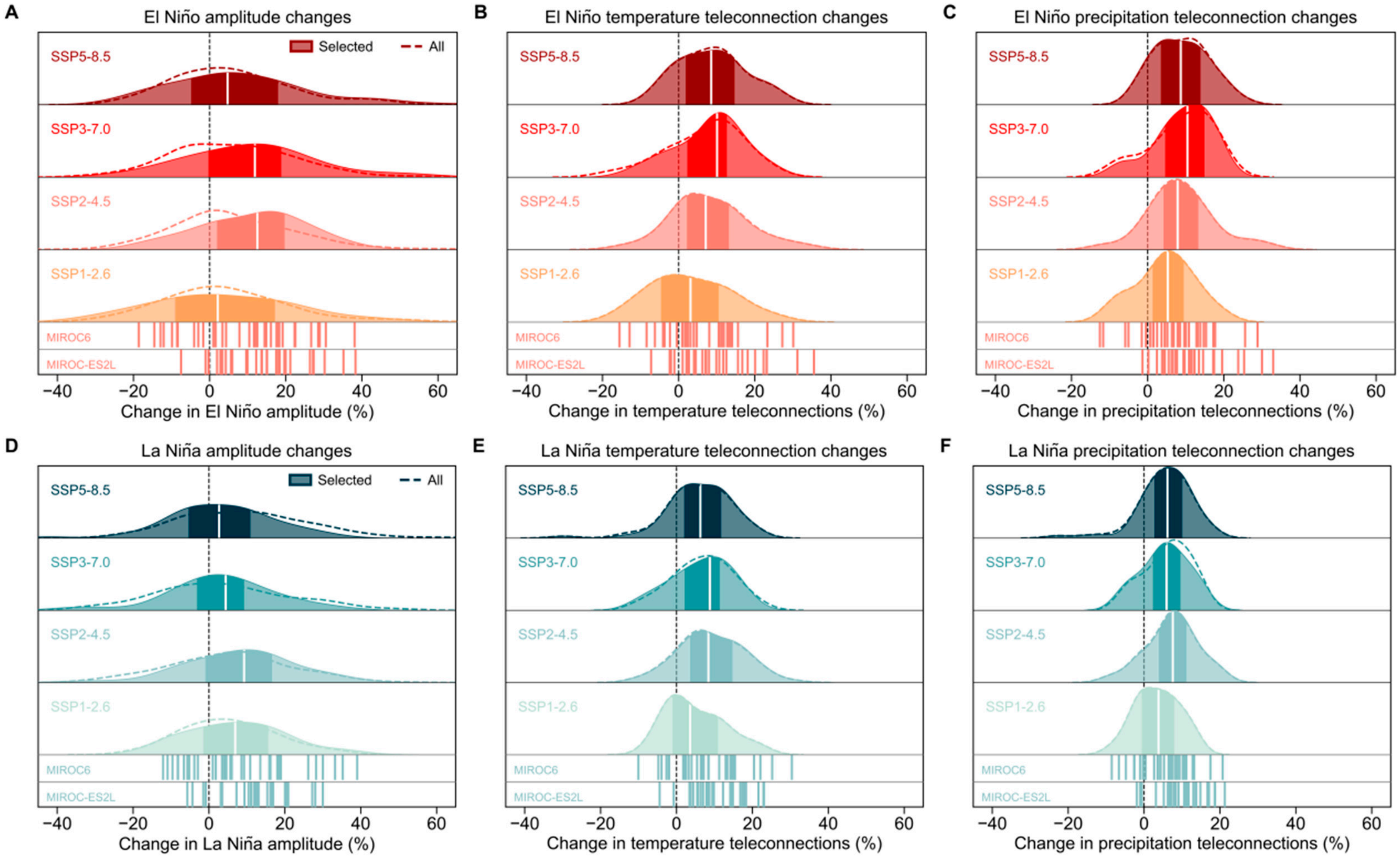

Figure S3. Projected changes in ENSO amplitude and teleconnection strength across SSP scenarios (2026–2090 relative to 1960–2024).

Panels A–C show ridge plots of the distribution of projected changes (%) in El Niño (E-index) amplitude (A), temperature teleconnection

strength (B), and precipitation teleconnection strength (C) across CMIP6 model realizations under four SSP scenarios. Panels D – F show the corresponding distributions for La Niña (C-index). Solid filled distributions represent model realizations retained after quality screening (Selected); dashed outlines represent all available realizations (All). White vertical lines indicate the median of the selected distribution. Rug plots at the bottom of each panel show individual realizations from MIROC6 and MIROC-ES2L. Positive values indicate projected strengthening relative to the historical baseline.

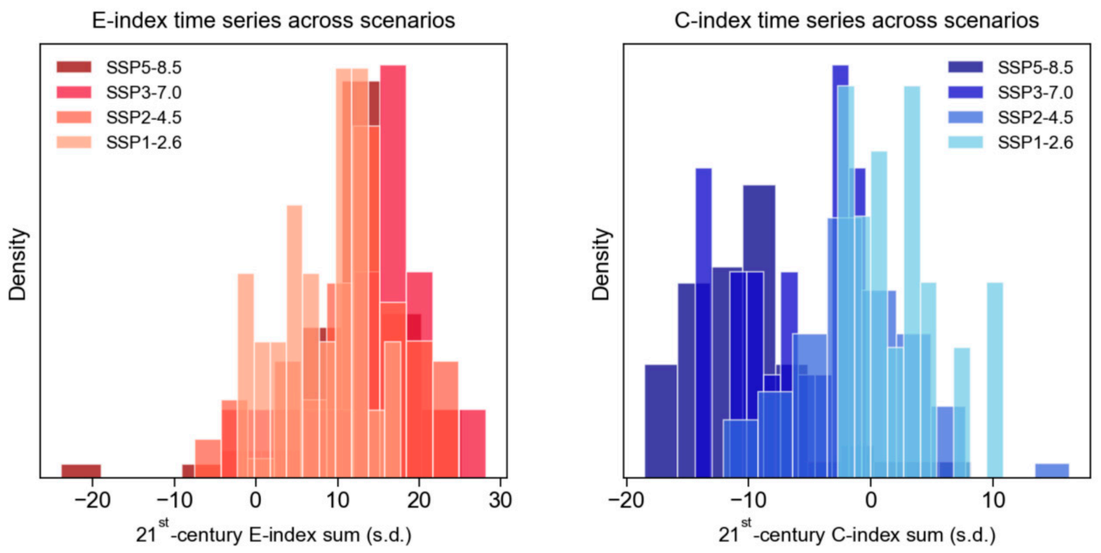

Figure S4. E-index and C-index sum across scenarios. The left panel shows histograms of the distribution of 2026–2090 E-index sum values across simulations within each SSP scenario. Positive values indicate that the simulated E-index time series contains more El Niños than La Niñas. The right panel shows histograms of the distribution of 2026–2090 C-index sum values across simulations within each SSP scenario. Negative values indicate that the simulated C-index time series contains more La Niñas than El Niños.

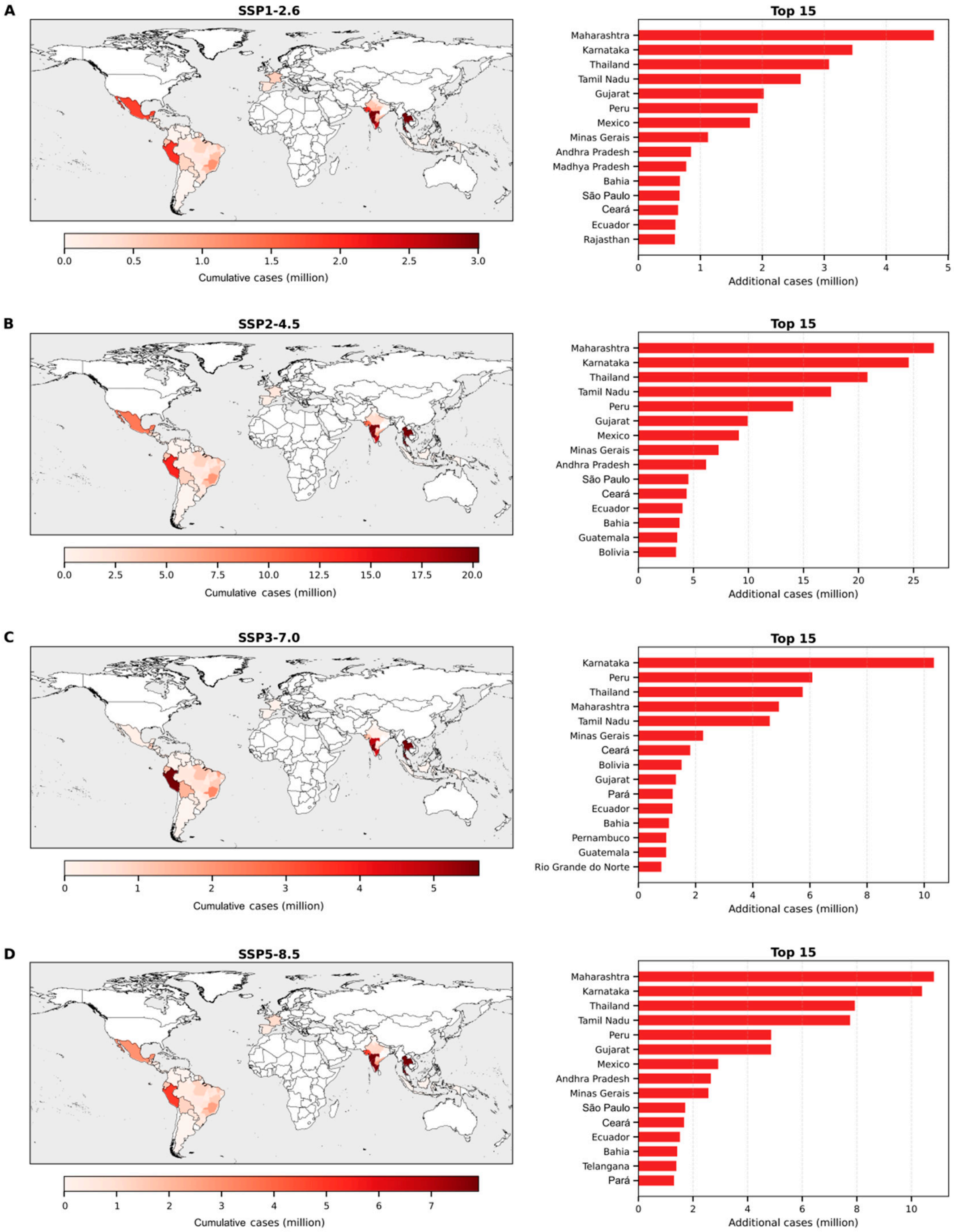

Figure S5. Projected additional chikungunya burden attributable to climate change-driven shifts in ENSO amplitude under four SSP scenarios (2030–2050). Maps show the cumulative additional chikungunya cases projected under SSP1-2.6 (A), SSP2-4.5 (B), SSP3-7.0 (C), and SSP5-8.5 (D) over 2030–2050. Positive values indicate additional cases attributable to the intensification of ENSO variability driven by global warming. Right panels display the 15 administrative units with the greatest projected additional burden.

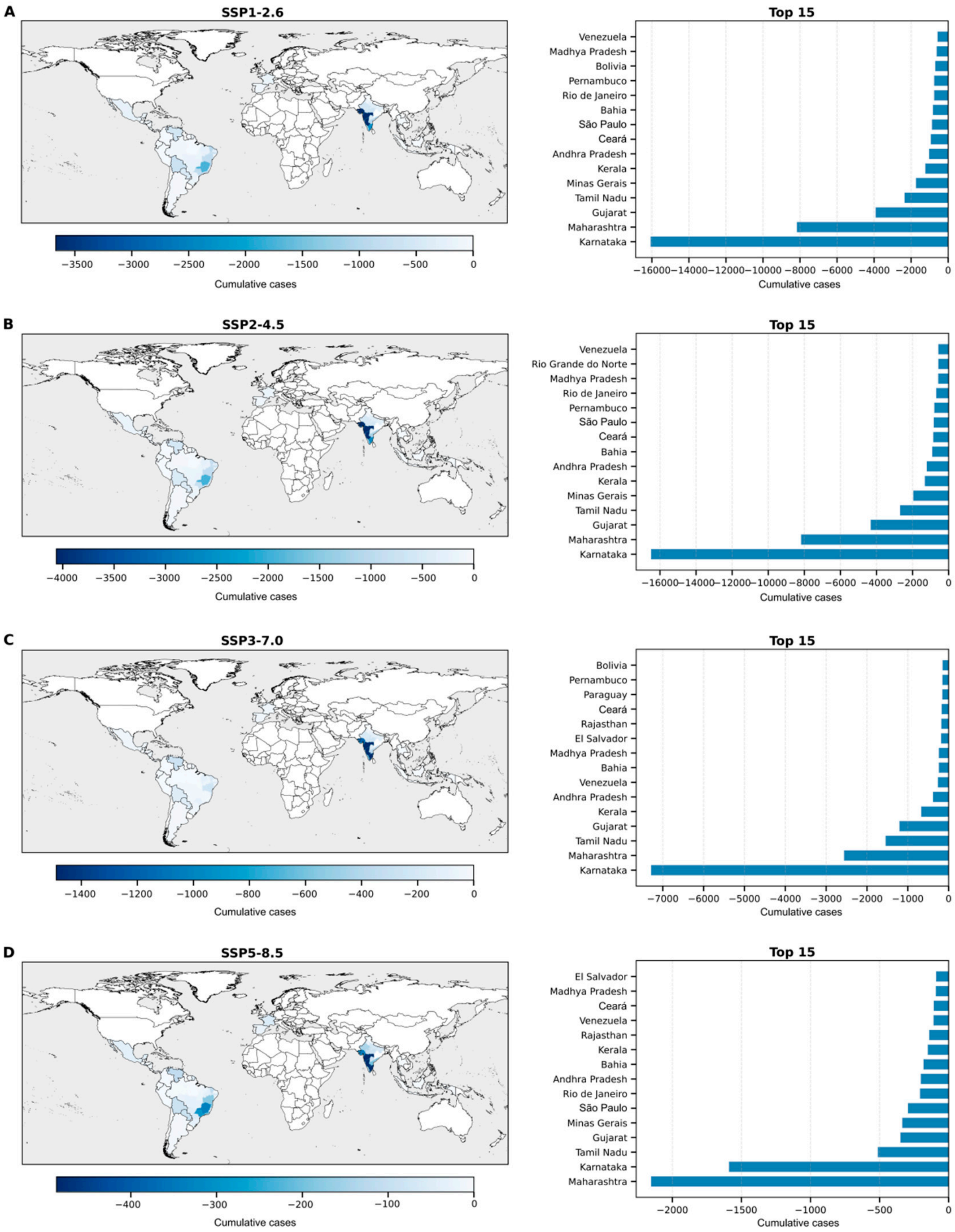

Figure S6. Projected averted chikungunya burden attributable to climate change-driven shifts in ENSO amplitude under four SSP scenarios (2030–2050). Maps show the cumulative reduction in chikungunya cases projected under SSP1-2.6 (A), SSP2-4.5 (B), SSP3-7.0 (C), and SSP5-8.5 (D) over 2030–2050. Negative values indicate averted cases attributable to changes in ENSO variability driven by global warming. Right panels display the 15 administrative units with the greatest projected reduction in burden.

Table S1. Chikungunya case data sources.

Geographic Coverage	Resolution	Year	Data Source	URL
Global	National	1991–2024	Global Infectious Disease and Epidemiology Online Network (GIDEON)	https://app.gideononline.com/start
Europe	National	2008–2025	European Centre for Disease Prevention and Control (ECDC)	https://atlas.ecdc.europa.eu/public/index.aspx?Dataset=27&HealthTopic=11 https://www.ecdc.europa.eu/en/chikungunya-virus-disease/surveillance-and-updates/seasonal-surveillance
Global	National	2011–2022	Global geotemporal distribution of chikungunya disease, 2011–2022	https://www.sciencedirect.com/science/article/pii/S1477893923000637
India	Subnational	2018–2025	National Vector Borne Disease Control Programme (NVBDCP)	https://ncvbdc.mohfw.gov.in
India	Subnational	2006, 2008, 2009, 2012, 2015–2019	Current Status of Chikungunya in India	https://pmc.ncbi.nlm.nih.gov/articles/PMC8274422/#S13
India	Subnational	2006, 2014, 2015	National Guideline for Clinical Management of Chikungunya	https://docslib.org/doc/799201/national-guidelines-for-clinical-management-of-chikungunya-2016
India	Subnational	2017–2025	Global Infectious Disease and Epidemiology Online Network (GIDEON)	https://app.gideononline.com/start
Brazil	Subnational	1975–2025	DATASUS/TABNET, Brazilian Ministry of Health	https://datasus.saude.gov.br/informacoes-de-saude-tabnet/

Continued Table S1.

United States	Subnational	2016–2023	US Centers for Disease Control and Prevention (NNDSS)	https://wonder.cdc.gov/controller/datarequest/D130
Australia	Subnational	1991–2025	Australian National Notifiable Diseases Surveillance System (NNDSS)	https://nindss.health.gov.au/pbi-dashboard/

Table S2. Climate and Socioeconomic Data sources.

Category	Dataset	Resolution	Source
Climate data	Monthly mean sea surface temperatures	1°×1°	HadISST (https://www.metoffice.gov.uk/hadobs/hadisst/data/download.html)
	Monthly total precipitation data	0.25°×0.25°	ERA5 (https://cds.climate.copernicus.eu/datasets/reanalysis-era5-single-levels-monthly-means?tab=download)
	Monthly mean atmospheric temperatures	0.25°×0.25°	ERA5 (https://cds.climate.copernicus.eu/datasets/reanalysis-era5-single-levels-monthly-means?tab=download)
	Climate change projection data	0.25°×0.25°	The sixth phase of the Climate Model Intercomparison Project (https://esgf-node.llnl.gov/search/cmip6/)
Socioeconomic data	Historical population data (2008-2022)	30arc	GlobPOP (https://zenodo.org/records/11179644)
	Historical population data (2023-2025)	1km	WorldPop (https://hub.worldpop.org/geodata/listing?id=137)
	Future population data	1km	Scientific Data (https://figshare.com/articles/dataset/Projecting_1_km-grid_population_distributions_from_2020_to_2100_globally_under_shared_socioeconomic_pathways/19608594/2)

Table S3. Comparison of model performance at different case thresholds under a maximum lag of two years. The threshold refers to excluding countries with cumulative case counts below a specified threshold. *n* denotes the number of nations and sub-national areas included at each threshold. R^2(e): R^2 from models including the E-index and its temperature and precipitation teleconnections, R^2(c): R^2 from models including the C-index and its temperature and precipitation teleconnections. All models were fitted using optimal regression structures, with results demonstrating the explanatory power of each model at different thresholds.

Threshold	*n*	R^2(e)	R^2(c)
1000	73	0.54	0.46
500	77	0.49	0.41
100	95	0.48	0.43

Table S4. Correlation matrix for the E-index and its lags. Each table entry shows the Pearson correlation coefficient between the E-index at various time lags and the E-index at each other time lag.

	E_t	E_{t-1}	E_{t-2}	E_{t-3}
E_t		−0.069	−0.283	0.010
E_{t-1}			−0.076	−0.288
E_{t-2}				−0.089

Table S5. Correlation matrix for the C-index and its lags. Each table entry shows the Pearson correlation coefficient between the C-index at various time lags and the C-index at each other time lag.

	C_t	C_{t-1}	C_{t-2}	C_{t-3}
C_t		0.187	−0.012	0.061
C_{t-1}			0.178	−0.030
C_{t-2}				0.168

Table S6. Comparison of E-index model results accounting for the impact of the COVID-19 pandemic. The main model covers the period from 2008 to 2024 and includes the Oxford Stringency Index (OSI). The sensitivity analysis restricts the study period to 2008–2019 (pre-pandemic) and excludes the OSI. The table presents the coefficients for the E-index and interaction terms. Regression coefficients for the main model were calculated using the main regression model (Eqn. 3). Standard errors are indicated in parentheses. Country and region fixed effects are included in both models but are not listed in this table. ***p < 0.001; **p < 0.01; *p < 0.05

Variable	2008–2024	2008–2019
$E_t(\beta_0)$	−0.76291**	−0.85770
	(0.25521)	(0.50038)
$E_t^2(\beta_0)$	1.01456***	1.04185**
	(0.18410)	(0.32245)
$E_{t\text{-}1}(\beta_1)$	−1.45459***	−1.03362***
	(0.21544)	(0.27860)
$E_{t\text{-}1}^2(\beta_1)$	1.11646***	0.97606***
	(0.15650)	(0.17358)
$E_{t\text{-}2}(\beta_2)$	−0.65583**	−0.45183
	(0.22488)	(0.55965)
$E_{t\text{-}2}^2(\beta_3)$	0.36503*	0.24489
	(0.18038)	(0.50282)
$E_t{}^{\times}\tau^{E,T}{}_i(\theta_0)$	−0.25793	−2.07310
	(0.61182)	(1.20962)
$E_t{}^{2\times}\tau^{E,T}{}_i(\theta_0)$	0.06100	0.89066
	(0.44517)	(0.77949)

Continued Table S6.

$E_t{}^{\times}\tau^{E,P}{}_i(\theta_0)$	−0.17426<br>(0.52054)	−1.55002*<br>(0.67349)
$E_t{}^{2\times}\tau^{E,P}{}_i(\theta_0)$	1.47180***<br>(0.37795)	2.26873***<br>(0.41961)
$E_{t\text{-}1}{}^{\times}\tau^{E,T}{}_i(\theta_1)$	−0.19618<br>(0.53130)	−1.06244<br>(1.35288)
$E_{t\text{-}1}{}^{2\times}\tau^{E,T}{}_i(\theta_1)$	1.00379*<br>(0.43416)	0.46695<br>(1.21551)
$E_{t\text{-}1}{}^{\times}\tau^{E,P}{}_i(\theta_1)$	−0.21229<br>(0.54423)	0.87120<br>(1.07641)
$E_{t\text{-}1}{}^{2\times}\tau^{E,P}{}_i(\theta_1)$	0.51961<br>(0.39602)	0.08745<br>(0.69365)
$E_{t\text{-}2}{}^{\times}\tau^{E,T}{}_i(\theta_2)$	−0.67188<br>(0.46323)	0.34775<br>(0.59932)
$E_{t\text{-}2}{}^{2\times}\tau^{E,T}{}_i(\theta_3)$	0.97707**<br>(0.33632)	0.71617<br>(0.37340)
$E_{t\text{-}2}{}^{\times}\tau^{E,P}{}_i(\theta_2)$	−1.19296*<br>(0.47283)	−0.12342<br>(1.20389)
$E_{t\text{-}2}{}^{2\times}\tau^{E,P}{}_i(\theta_3)$	0.87732*<br>(0.38626)	0.90406<br>(1.08165)
OSI(α)	0.03528***<br>(0.00410)	—
Adjusted R-squared	0.504	0.523
BIC	6286.38	4404.39
Residual Standard Error	2.424	2.212

Table S7. Comparison of C-index model results accounting for the impact of the COVID-19 pandemic. The main model covers the period from 2008 to 2024 and includes the Oxford Stringency Index (OSI). The sensitivity analysis restricts the study period to 2008–2019 (pre-pandemic) and excludes the OSI. The table presents the coefficients for the C-index, and interaction terms. Regression coefficients for the main model were calculated using the main regression model (Eqn. 3). Standard errors are indicated in parentheses. Country and region fixed effects are included in both models but are not listed in this table. ***$p < 0.001$; **$p < 0.01$; *$p < 0.05$

Variable	2008-2024	2008-2019
$C_t (\beta_0)$	−0.16932 (0.21594)	0.12471 (0.22866)
$C_t^2 (\beta_0)$	−0.65276** (0.19827)	−0.40192* (0.18142)
$C_{t-1} (\beta_1)$	−0.08778 (0.23749)	0.65795** (0.23196)
$C_{t-1}^2 (\beta_1)$	−0.33778 (0.20304)	0.07057 (0.18183)
$C_{t-2} (\beta_2)$	0.26407 (0.26340)	0.54428* (0.24031)
$C_{t-2}^2 (\beta_3)$	−0.37026 (0.19816)	0.20580 (0.18344)
$C_t \times \tau^{C,T}_i (\theta_0)$	0.91692* (0.45513)	0.80414. (0.48216)
$C_t^{2} \times \tau^{C,T}_i (\theta_0)$	0.00142 (0.41747)	−0.28530 (0.38253)

Continued Table S7.

$C_t{}^{\times}\tau^{C,P}{}_i(\theta_0)$	0.87083<br>(0.50019)	0.02329<br>(0.48910)
$C_t{}^{2\times}\tau^{C,P}{}_i(\theta_0)$	−0.17799<br>(0.42808)	−0.64138.<br>(0.38340)
$C_{t\text{-}1}{}^{\times}\tau^{C,T}{}_i(\theta_1)$	0.46040<br>(0.55525)	0.21503<br>(0.50672)
$C_{t\text{-}1}{}^{2\times}\tau^{C,T}{}_i(\theta_1)$	0.13390<br>(0.41709)	−0.72580<br>(0.38680)
$C_{t\text{-}1}{}^{\times}\tau^{C,P}{}_i(\theta_1)$	−0.02243<br>(0.27025)	0.03497<br>(0.28632)
$C_{t\text{-}1}{}^{2\times}\tau^{C,P}{}_i(\theta_1)$	0.00049<br>(0.24782)	0.07852<br>(0.22716)
$C_{t\text{-}2}{}^{\times}\tau^{C,T}{}_i(\theta_2)$	0.02384<br>(0.29704)	0.36419<br>(0.29044)
$C_{t\text{-}2}{}^{2\times}\tau^{C,T}{}_i(\theta_3)$	0.00808<br>(0.25421)	0.16503<br>(0.22767)
$C_{t\text{-}2}{}^{\times}\tau^{C,P}{}_i(\theta_2)$	0.09265<br>(0.32958)	0.25459<br>(0.30091)
$C_{t\text{-}2}{}^{2\times}\tau^{C,P}{}_i(\theta_3)$	−0.10621<br>(0.24755)	0.15967<br>(0.22969)
OSI(α)	0.00944*<br>(0.00420)	—
Adjusted R-squared	0.414	0.433
BIC	6492.996	4440.927
Residual Standard Error	2.634	2.259

Table S8. CMIP6 models and realizations selected for predicting future infectious disease risks for each of the four Shared Socioeconomic Pathways scenarios (SSP1-2.6, SSP2-4.5, SSP3-7.0 and SSP5-8.5). Monthly sea surface temperature (“tos”), monthly atmospheric temperature (“tas”), and daily precipitation (“pr”) are extracted from each realization for prediction.

Scenarios	Model	Total # of Realizations	# of Realizations Selected for Prediction
SSP1-2.6	MIROC-ES2L	13	7
	MIROC6	24	24
	MPI-ESM1-2-HR	36	0
	NorESM2-MM	34	0
	CESM2-WACCM	5	0
	CMCC-CM2-SR5	1	0
SSP2-4.5	MIROC-ES2L	30	29
	MIROC6	33	33
	CESM2-WACCM	10	2
	CMCC-CM2-SR5	1	1
	MPI-ESM1-2-HR	36	1
	NorESM2-MM	18	1
SSP3-7.0	MIROC-ES2L	10	10
	MIROC6	50	3
	CMCC-CM2-SR5	1	1
	CESM2-WACCM	4	0
	MPI-ESM1-2-HR	180	4
	NorESM2-MM	9	1
SSP5-8.5	MIROC-ES2L	11	9
	MIROC6	50	50
	MPI-ESM1-2-HR	36	1
	NorESM2-MM	9	1
	CESM2-WACCM	7	1
	CMCC-CM2-SR5	1	1
Total		609	180
